# Phosphatidylcholine formation by LPCAT1 is regulated by Ca^2+^ and the redox status of the cell

**DOI:** 10.1186/1471-2091-13-8

**Published:** 2012-06-07

**Authors:** Eric Soupene, Frans A Kuypers

**Affiliations:** 1 Children’s Hospital Oakland Research Institute, 5700 Martin Luther King Jr. Way, Oakland, CA, 94609, USA

**Keywords:** Lands’ cycle, Cysteine oxidation, Calcium binding, Plasma membrane

## Abstract

**Background:**

Unsaturated fatty acids are susceptible to oxidation and damaged chains are removed from glycerophospholipids by phospholipase A_2_. De-acylated lipids are then re-acylated by lysophospholipid acyltransferase enzymes such as LPCAT1 which catalyses the formation of phosphatidylcholine (PC) from lysoPC and long-chain acyl-CoA.

**Results:**

Activity of LPCAT1 is inhibited by Ca^2+^, and a Ca^2+^-binding motif of the EF-hand type, EFh-1, was identified in the carboxyl-terminal domain of the protein. The residues Asp-392 and Glu-403 define the loop of the hairpin structure formed by EFh-1. Substitution of D^392^ and E^403^ to alanine rendered an enzyme insensitive to Ca^2+^, which established that Ca^2+^ binding to that region negatively regulates the activity of the acyltransferase amino-terminal domain. Residue Cys-211 of the conserved motif III is not essential for catalysis and not sufficient for sensitivity to treatment by sulfhydryl-modifier agents. Among the several active cysteine-substitution mutants of LPCAT1 generated, we identified one to be resistant to treatment by sulfhydryl-alkylating and sulfhydryl-oxidizer agents.

**Conclusion:**

Mutant forms of LPCAT1 that are not inhibited by Ca^2+^ and sulfhydryl-alkylating and –oxidizing agents will provide a better understanding of the physiological function of a mechanism that places the formation of PC, and the disposal of the bioactive species lysoPC, under the control of the redox status and Ca^2+^ concentration of the cell.

## Background

The oxygen carrying function of the red blood cell (RBC) leads to the generation of reactive oxygen species in the cell, and despite an intricate system of antioxidants, free radical damage of un-saturated acyl chains of glycerophospholipids occurs continuously. These oxidized acyl chains lead to a breach in normal membrane lipid organization and need to be replaced to maintain integrity of the membrane. The oxidized phospholipids (PL) are de-acylated by phospholipase A_2_ (PLA_2_) action [1-4], and re-acylation of the resulting lysophospholipid (lysoPL) is achieved by a two-step process. Fatty acids are activated to acyl-CoAs by membrane-bound long-chain acyl-CoA synthetases (ACSL) [5-7] and the acyl group of acyl-CoA is then transferred to lysoPL by acyl-CoA:lysoPL acyltransferase (LPLAT) enzymes [8-11]. This repair mechanism is also known as the Lands’ cycle [8,12].

Phosphatidylcholine (PC) is the most abundant glycerophospholipid in membranes [13], with unsaturated acyl chains, mainly found at the sn-2 position. Repair of oxidized PC and re-acylation of the lysoPC in RBC proceeds rapidly by utilizing fatty acids that are taken up from plasma, and the action of ACSL and LPCAT in the plasma membrane [14]. We previously identified ACSL6 as the acyl-CoA synthetase in the RBC membrane and LPCAT1 as the acyl-CoA:lysoPC acyltransferase [7,15-17]. LPCAT1 is also the enzyme for the re-acylation of PC in alveolar type II cells [18,19]. Furthermore, LPCAT1 might play an essential role in production of lipid surfactant in lung [20,21], and in regulating the level of inflammatory lipids, such as lysoPAF and lysoPC, in the retina [22,23]. LPCAT1 also appears to mediate O-palmitoylation of histone H4 in the nuclei of lung epithelial cell [24].

LPCAT1 does not require Ca^2+^ for activity [16,19] and was reported as a Ca^2+^-independent member of the LPCAT family of enzymes [25-27]. Activity of LPCAT2 is regulated by Ca^2+^[16,25,28] and was defined as a Ca^2+^-dependent member of the LPCAT family [25-28]. However, two EF-hand motifs, folding into hairpin structure coordinating Ca^2+^[29-32], are predicted in both LPCAT1 and LPCAT2 [16,18,28]. Although, we have confirmed that Ca^2+^was not required for activity of LPCAT1, i.e. Ca^2+^-independent in [25], we have established that Ca^2+^was in fact inhibitory on the LPCAT1 activity [16]. At the millimolar Ca^2+^ concentration values found in plasma [33], acylation rate of LPC by LPCAT1 was reduced and showed dependency to Ca^2+^ concentration [16]. Thus, as it is the case for LPCAT2, Ca^2+^ also regulates the activity of LPCAT1. These observations led us to investigate the role of the predicted EF-hand motifs in Ca^2+^-binding.

LPCAT1 activity is also sensitive to treatment by sulfhydryl-modifier agents, such as the alkylating thiol reductant N-ethyl maleimide (NEM) [34]. Cursory observation indicated that of the 12 cysteines of LPCAT1, Cys-211 found at the +1 position of motif III, ^207^PEGT^210^, could be conserved among acyltransferase forms that are sensitive to NEM and may be responsible for their sensitivity to this agent [34]. This residue was also proposed to define the ‘motif 3-cysteine acytransferases’ sub-family of LPLAT enzyme and to be crucial for catalysis [34]. However, the role of Cys-211 in the sensitivity to NEM and in catalysis was never tested since even the substitution of Cys-211 to the arginine residue present at the end of motif III of LPAAT enzymes [35], rendered an inactive C^211^R form [34]. Similarly, substitution of Arg-181 of motif III of the human LPAAT enzyme AGPAT1 to several other residues rendered inactive forms [36].

We report that the EFh-1 motif of LPCAT1 is a functional Ca^2+^-binding site and that Cys-211 is not essential for activity of LPCAT1. Up to six cysteines residues, including Cys-211, are responsible for the decrease activity of the enzyme after treatment by NEM and diamide. The sensitivity of LPCAT1 activity to thiol damage and to Ca^2+^ binding to the EFh-1 site establishes that acylation of the most abundant phospholipids of the cell membranes is under the control of the redox status and Ca^2+^ concentration of the cell.

## Results

### Role of Asp-392 and Glu-403 residues in calcium inhibition

**Figure 1:**
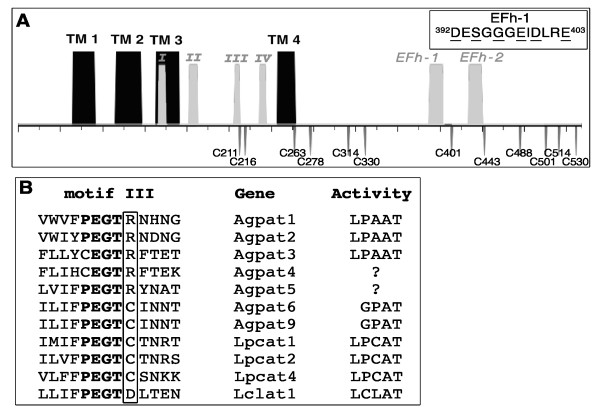
**Cartoon representation of LPCAT1 (A) and alignment of Motif III residues of some LPLAT members (B). (A)** Motifs I, II, III and IV of LPCAT1 enzyme (GenBank accession number: NP_663351), conserved among LPLAT enzymes, are indicated in light grey. Four predicted transmembrane spanning segments (TM1, TM2, TM3, TM4) [16] are shown in black. The topology of LPCAT1 is not know and the presented model is only representative of a prediction that would support orientation of all 4 conserved motifs to the cytosolic side (or near the cytosolic membrane surface) with exposure of the two predicted EF-hand motifs on the cell surface. The amino acid sequence of the EFh-1 motif, with the 6 most conserved residues underlined, is shown. The 12 cysteines are also indicated. **(B)** Amino acid alignment of motif III of members of the mouse Agpat and Lpcat families. The arginine and a cysteine residue found at the + 1 position of the PEGT motif are indicated. When known, enzymatic activity of the protein is indicated on the right. Substrate specificity of Agpat4 and Agpat5 is not known but the two members are predicted to be LPAAT enzyme. Agpat7 is now annotated as Lpcat4. The acyl-CoA:lysoCardiolipin acyltransferase 1 protein (ALCAT1) [42], currently annotated as LCLAT1, was mis-identified by Agarwal *et al*. [43] as a new member of the AGPAT family, AGPAT8. The annotation for Agpat8 has been removed from database. Not shown is LPCAT3, formerly annotated as membrane-bound O-acyltransferase 5 (Mboat5), which differs from other LPCAT members [44,45].

We previously determined that activity of mouse LPCAT1 (formerly known as Aytl2) and of LPCAT2 (Aytl1) were inhibited in presence of millimolar concentration of calcium chloride. Addition of EDTA to the reaction mixture restored activity [16]. As observed in some membranes [9,37], physiological intracellular Ca^2+^ concentration (< 0.1 mM) had no effect on activity. The activity of third member Aytl3, currently annotated as LPCAT4, was not affected by the presence of Ca^2+^. A pair of Ca^2+^-binding sites of the EF-hand type was predicted at the carboxy-terminal extremity of LPCAT1 and LPCAT2 but not of LPCAT4 ([16] and Figure 1A). Of the two motifs, EFh-1 was the best match to a consensus sequence (^392^DxSxGxExDx_2_E^403^), as it contains the crucial first aspartate and last glutamate residues, which are necessary to coordinate the divalent cation to the hairpin structure [31,38-41].

**Figure 2:**
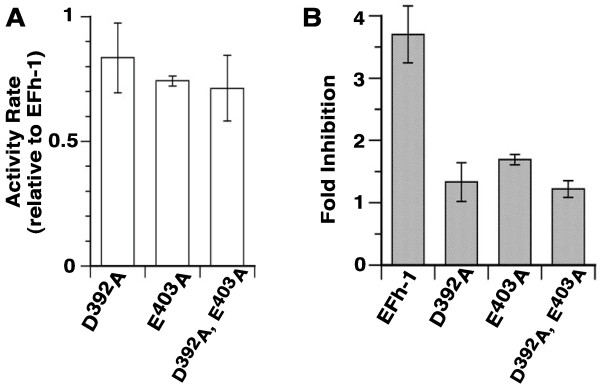
**Role of D**^**392**^**and E**^**403**^**in Ca**^**2+**^**-inhibition of LPCAT1 activity.** Asp-392 and Glu-403 of the predicted EFh-1 motif of LPCAT1 were changed by site-directed mutagenesis to an alanine, and the activity of the three mutants enzymes, D^392 ^ A, E^403 ^ A and D^392^A,E^403^A was determined. Activity measurements were performed with 5M [^14 ^ C]-C_18:1 _ -CoA in the presence of 20 μM LPC at 37°C with 1μg of proteins. Acylation rates were calculated between 0 and 8 min. The standard deviation of 3 different measurements is indicated as error bars. After separation by thin-layer chromatography, the amount of [^14^C]-PC formed during the reaction was quantified by phosphoimaging (see the Experimental section) and the activity rate values are calculated as PC formed/ μg of protein per min. **(A)** Measurement of the activity rates was performed with LPCAT1 and the 3 mutant enzymes. Values obtained for the mutant forms are reported relative to the activity rate value obtained with LPCAT1. **(B)** Activity of LPCAT1 and of the three mutant forms was determined in the absence and in the presence of 10 mM calcium chloride. Activity rates were calculated as described above. The ratio of the value obtained in presence of Ca^2+^ relative to the value obtained in its absence for each protein is reported as fold inhibition. Note that a ratio value of 1 indicates that the activity rate in presence and in absence of Ca^2+^ was identical.

Asp-392 and Glu-403 were substituted by site-directed mutagenesis to alanine. The two single-substituted recombinant protein, D^392^A and E^403^A, and the double mutant form, D^392^A/E^403^A, were produced and their activities in absence and presence of an inhibitory Ca^2+^ concentration of 10 mM [16] were determined. All three mutants proteins were active and their activity rate was 70 to 80% of LPCAT1 activity (Figure 2A). Thus, Asp-392 and Glu-403, which are not conserved in LPLAT enzymes, are dispensable for activity. Activity of the mutant forms was less sensitive to presence of Ca^2+^ than LPCAT1. In particular, activity of the double mutant form D^392^A/E^403^A was unaffected even in presence of 10 mM calcium chloride (the highest concentration tested), which resulted in a 4-fold inhibition of activity of LPCAT1 (Figure 2B). These findings establish that EFh-1 motif is a functional Ca^2+^-binding site. It was reported that LPCAT1 does not require Ca^2+^[19,25-27], These data show that the activity of LPCAT1 is regulated by high Ca^2+^-concentrations.

### Sensitivity of LPCAT1 to thiol alkylation and oxidation

**Figure 3:**
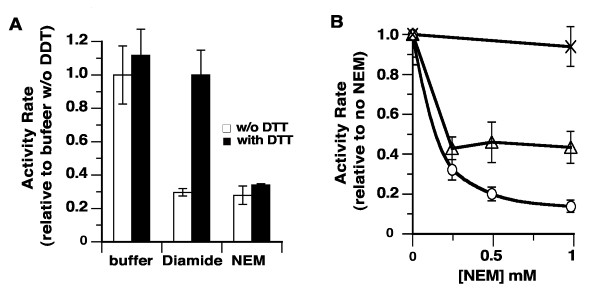
**Effect of sulfhydryl-alkylation and of disulfite bond formation on activity.** Measurement of LPCAT1 activity was performed as described in legend of Figure 2. The standard deviation of at least 3 different measurements is indicated as error bars. **(A)** Treatment with diamide (0.5 mM) and N-ethylmaleimide (0.5 mM) were performed for 30 min before assaying acyltransferase activity. In some reactions, DTT was added at a concentration of 20 mM after treatment with diamide or NEM, and mixtures were incubated for another 10 min. Concentrations of the reagents were reduced 20-fold by dilution into the acyltransferase reaction mixture to assay the activity of the treated enzyme. Before assaying their activity, control samples were incubated for the same period of time and under the same condition in absence of diamide, of NEM and of DTT. The activity rate value obtained in absence of any of these chemicals (buffer without DTT) was arbitrary set at 1 and all others values were calculated relative to it. **(B)** Activity rate of LPCAT1 (circle) and of mutant C^211^T (triangle) were measured after treatment by different concentrations of NEM, as described above. For each protein, the value obtained in absence of treatment was set at 1 and values obtained after treatment were calculated relative to it. The activity rate ratio of the mutant form C^211^T, C^(216,314,443,501,514)^A obtained without and after treatment with 1 mM NEM is also reported (cross symbol).

LPCAT1 has 12 cysteine residues and the activity of human LPCAT1 was shown to be inhibited by the thiol alkylating agent N-ethylmaleimide (NEM) [34]. Sensitivity of the LPCAT1 acyltransferase activity to oxidation of thiol groups of cysteines was determined after incubation with either NEM or the cross-linker agent diamide at a concentration of 0.5 mM for 30 minutes. Subsequently the mixtures were diluted 20-fold and activity was measured. Under these conditions, LPCAT1 activity was reduced to 20-25% as compared to the activity after incubation with buffer (Figure 3). In addition, protein was also labeled by a biotinylated-version of NEM, 3-maleimidylpropionyl-biocytin (MPB), which confirmed the sensitivity of some sulfhydryl groups to the alkylating agent (see below). Treatment of LPCAT1 with 20 mM DTT had little effect on activity suggesting that in the membrane, cysteines important for activity were in a reduced state (Figure 3A, first filled bar). Diamide generates disulfide bonds between cysteines whereas NEM alkylates -SH group of cysteines. As anticipated, incubation with DTT fully restored activity of a diamide-treated enzyme mixture but the covalent modification of these cysteines by NEM could not be reversed by DTT treatment (Figure 3A).

### Cys-211 is not essential for activity

**Figure 4:**
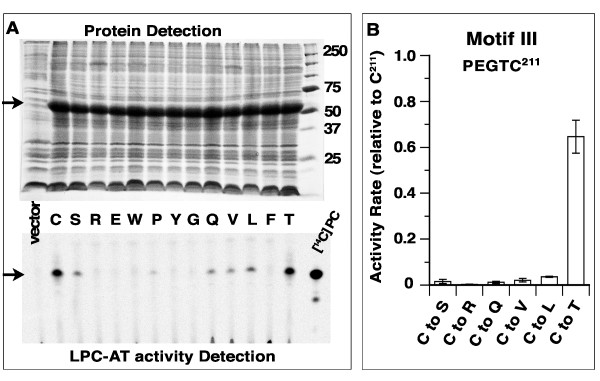
**Cys-211 is not essential to the acyltransferase reaction.** A collection of mutant forms in which C^211^ was changed to E, W, P, Y, G, Q, V, L, F and T was obtained by random mutagenesis of the Cys-211 codon. In addition, site-directed mutagenesis was performed to change C^211^ to S and R. Clones were tested for production of the mutant protein [panel A, top gel] and for their lysoPC-acyltransferase activity [panel A, bottom gel]. **(A)***Top gel:* Proteins were separated on a 12% gel SDS-PAGE and stained with coomassie blue. Molecular mass standard (Precision Plus Protein Standard, Bio-Rad) is shown on the right (last lane). Position of the LPCAT1 protein (lane C) and of the mutant forms (S, R, E, W, P, Y, G, Q, V, L, F, T) is indicated with a black arrow on the left. Note the absence of LPCAT1 protein in the control sample prepared from cells containing the empty expression vector (lane vector). Production and position of the proteins were confirmed by Western blot detection of the hexa-histidine tags with an anti-histidine antibody (India-His, Pierce). **(A)***Bottom gel:* Detection of the lysoPC-acyltransferase activity was performed with 5 μM [^14^C]-C_18:1_-CoA in presence of 20 μM LPC at 37°C with a 20 min incubation and with 10 μg of proteins, which represent 10 time more enzyme than the amount used to calculate activity rate (1 μg). Products were separated on silica plates and detected by phosphoimaging. Pure [^14^C]-PC was used as a migration standard, last lane on the right and position of [^14^C]-PC is indicated on the left with an arrow. Note the absence of the product [^14^C]-PC in the vector lane (left). **(B)** Activity rate measurements were performed as described in legend of Figure 2. LPCAT1, Rates of C^211^R mutant (see text) and of the C^211^ mutant forms able to form [^14^C]-PC, as shown on panel A, were determined. Values obtained for the mutant forms are reported relative to the activity rate obtained with the LPCAT1 enzyme (dubbed C^211^). Amino acid sequence of motif III is shown. The standard deviation of at least 3 different measurements is indicated as error bars.

In order to determine the role of Cys-211 (Figure 1A) in catalysis and in sensitivity of the enzyme to NEM treatment, we performed a saturated random mutagenesis of the Cys-211codon and screened the collection of clones for active mutant forms (Table 1). As observed previously [34], we confirmed that substitution of Cys-211 to most other residues greatly reduced activity of LPCAT1, resulting in enzymes with low or no detectable activity (Figure 4). However, one mutant form, C^211^T, could produce as much PC as LPCAT1 (Figure 4A), with an activity rate of about 60% of LPCAT1 (Figure 4B). Thus, Cys-211 is not essential for catalysis.

**Table 1:** List of primers used for mutagenesis

**Purpose**	**Primer sequence (5′ to 3′)**
saturated mutagenesis of	GCCTCAGATAATGATTTTTCCAGAAGGAACTNNKACAAA-
Cys-211 codon	TAGGACCTGCCTCA
Cys-211 to Ser	GATTTTTCCAGAAGGAACTAGTACAAATAGGACCTGCCT
Glu-403 to Ala	TGAGATTGACCTTCGTGCATATGTGGTCGCCTTGT
Cys-216 to Ala	GAACTGCTACAAATAGGACCGCCCTCATTACCTTCAAACCTG
Cys-263 to Ala	AATCCTGTGGCTCACTCTGGCCCAGTTTCAAAACCAAGTG
Cys-278 to Ala	GTGGAAATTGAATTTCTGCCTGTGTATGCCCCTTCTGAAGAGG
Cys-314 to Ala	ACTATACATTTGAGGACGCCCAGCTGGCTCTGGCAG
Cys-330 to Ala	CTTGCCTGCTGACACCGCCCTGCTAGAGTTTGCC
Cys-401 to Ala	GCCTTGTCTGTGGTGGCCAGGCCATCCCAGAC
Cys-443 to Ala	GAGGCCAACCTGTCCGCCATCCTCAAGACTGC
Cys-477 to Ala	AATCACCTTTGATGACTTCGCCGGGTTTGCGGAAATGTAC
Cys-501 to Ala	GACACATTTCGACAGCGCTGCACAGACACCCCCA
Cys-514 to Ala	CAACTCCCAATGGCTTCGCCATTGACTTCAGCCCTG
Cys-530 to Ala	ACTTTGGGAGAAAGAATTCTGCTAAGAAAGCGGACTAGCCTC

### Cys-211 is accessible to sulfhydryl-modifier reagents

**Figure 5:**
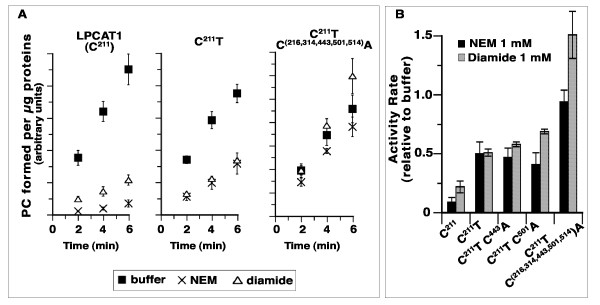
**Cysteine residues implicated in sensitivity to sulfhydryl-modifier agents.** Activity rate measurements were performed as described in the legend of Figure 2. Treatments with NEM and diamide were performed as described in legend of Figure 3. The standard deviation of at least 3 different measurements is indicated as error bars. Results obtained for mutant forms that are not shown here (clone FK480, FK481), are presented in Additional file 1: Figure S1A. **(A)** Activities of LPCAT1, C^211^T and of the mutant C^211^T, C^(216,314,443,501,514)^A in absence (filled square) and after treatment by 1 mM NEM (crosses) or by 1 mM diamide (triangles) are shown. Values are reported as amount of PC formed per μg of proteins in function of time. **(B)** Activity rates were measured as shown in panel A. For each protein, the value obtained in absence of treatment (buffer) was set at 1 and values obtained after treatments were calculated relative to it.

The rate of LPCAT1 activity is dependent on the concentration of NEM and was reduced almost ten-fold after treatment with 1 mM (Figure 3B, circle symbols). Although, the C^211^T protein was not as sensitive as LPCAT1 to NEM, it was still inhibited by this sulfhydryl reagent (Figure 3B). At a NEM concentration of 0.25 mM, the C^211^T enzyme was inhibited nearly as much as LPCAT1 (2.5-fold versus 3-fold). However, whereas treatment with higher NEM concentration (from 0.25 to 1 mM) decreased the activity rate of LPCAT1 another 3-fold, it had no additional inhibitory effect on the C^211^T form. Similarly, this mutant form was also less sensitive to inhibition by diamide (Figure 5A) as compared to LPCAT1. Thus, removal of Cys-211 had a protective effect towards the treatment by sulfhydryl-oxidizer and alkylating agents, but the biphasic pattern of the NEM titration of C^211^T activity also indicated that other cysteines altered catalysis after attack by the alkylating agent (Figure 3B). In support of these findings, we found that the C^211^T protein was still labeled by the biotinylated alkylating agent maleimidylpropionyl-biocytin (MPB) (see below).

### Role of cysteine residues in sensitivity to sulfhydryl modifier agents

**Figure 6:**
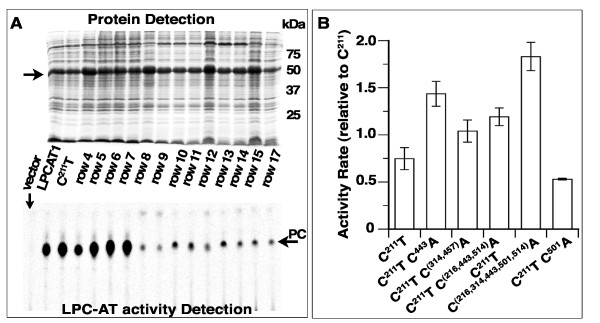
**Activity of Cysteine substitution mutant enzymes.** Alanine scanning of the 11 cysteines present in the active form C^211^T was performed to generate a collection of C-to-A mutant forms. The list of clones and their mutations are presented in Additional file 2: Table S1. **(A)** Clones were tested for production of the mutant protein (top gel) and for their lysoPC-acyltransferase activity (bottom) as described in the legend of Figure 4A. The row number (4 to 17) indicates the clone listed (row 4 to 17) in Additional file 2: Table S1. **(B)** Activity rate measurements were performed as described in the legend of Figure 4B. In addition to LPCAT1 and C^211^T, rate values for the mutant enzymes with significant acyltransferase activity as shown on panel A, [C^211^T C^443^A, row 4], [C^211^T C^(314,457)^A, row 5], [C^211^T C(^216,443,514^)A, row 6] and [C^211^T C^(216,314,443,501,514)^A, row 7], were determined. Protein expression and activity of the form C^211^T, C^501^A are presented in Additional file 1: Figure S1. Values are reported relative to the activity rate obtained with LPCAT1 enzyme. The standard deviation of at least 3 different measurements is indicated as error bars.

To identify other cysteine residues conferring sensitivity to NEM and diamide, we performed alanine scanning of the 11 cysteines still present in the active mutant form C^211^T. The mutants were generated by a multi-sites-directed mutagenesis approach to create a collection of different proteins with an increased probability to find active forms among them (see the Experimental section). The collection is listed in Additional file 2: Table S1. Protein expression, acyltransferase activity and sensitivity to sulfhydryl modifier agents of the mutant forms are shown in Figure 5, Figure 6 and Additional file 1: Figure S1.

The majority of the mutants displayed a very low activity (Figure 6A and Additional file 1: Figure S1). However in addition to the C^211^T form described above, five mutants missing 2 to 6 cysteines were obtained that showed acyltransferase activity ( Additional file 2: Table S1 and Figure 6). These 6 active proteins establish that Cys-211, 216, 314, 443, 501 and 514 are not essential to the acyltransferase reaction ( Additional file 2: Table S1). We could not identify a pattern and determine which cysteine, if any, was essential for activity. As observed for many of the substitutions tested with the Cys-211 residue and resulting in inactive mutant forms, alanine substitution of other non-essential cysteines could render mutant proteins with low activity due to structural alterations and defects. Among the active mutants, the form lacking all six cysteines mentioned above, C^211^T C^(216,314,443,501,514)^A, was the most active and had a higher activity rate than LPCAT1 ( Additional file 2: Table S1 and Figure 6). The other active forms displayed 50 to 150% of the activity of LPCAT1 (Figure 6). With one exception, all active forms were as sensitive to NEM and diamide treatment as was C^211^T (Figure 5B and Additional file 1: Figure S1). Activity of the mutant C^211^T C^(216,314,443,501,514)^A was not affected by treatment with either reagent (Figure 5). Even under condition resulting in 80 to 90% inhibition of the activity of LPCAT1, this mutant protein was still fully active. Moreover, it reproducibly displayed greater activity after treatment by diamide (Figure 5B, last bars).

### Cysteines labeling by maleimidylpropionyl-biocytin reagent

**Figure 7:**
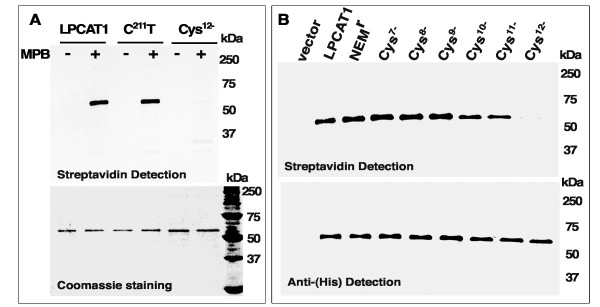
**Detection of sulfhydryl-modification of cysteine mutant forms.** Purified proteins (panel A) and membrane fractions obtained from cells producing LPCAT1 and several Cysteine mutant forms (panel B) were treated with maleimidylpropionyl-biocytin (MPB) as described in the Experimental section. **(A)** Proteins were purified and 2 μg aliquots were labeled with 100 μM of MPB protected from light. Reactions were stopped by quenching the unreacted MPB reagent with 25 mM DTT. Labeled and unlabeled proteins were separated on a 12% gel SDS-PAGE, and visualized by coomassie blue staining (bottom gel). Proteins were transferred onto PVDF membranes and blotted with HRP-conjugated streptavidin, which interacts with the biotin group of MPB. Molecular mass standard is shown on the right. **(B)** An equivalent of 25 μg of microsomal proteins as shown in Figure 4A and Figure 6A were labeled with 100 μM MPB. After quenching with DTT, hexa-histidine tagged LPCAT1 and mutant forms were affinity-purified. Proteins were separated on a 12% gel SDS-PAGE, transferred onto PVDF membranes and blotted with HRP-conjugated streptavidin (top membrane) or with a HRP-conjugated anti-histidine antibody (bottom). The mutant form lacking all 12 cysteines (Cys^12-^) was detected (last lane, bottom) but was not labeled (last lane, top). The mutant form C^211^T, C^(216,314,443,501,514)^A, whose activity was not inhibited by treatment with NEM , lane NEM^r^, was labeled by MPB. Results obtained with clone FK605, FK607, FK606, FK609, FK613 and FK614 (see Additional file 2: Table S1) are shown in lane Cys^7-^, Cys^8-^, Cys^9-^, Cys^10-^, Cys^11-^ and Cys^12-^, respectively. Molecular mass standard is indicated on the right.

LPCAT1, C^211^T and a mutant form lacking all 12 cysteines (Cys^12-^) were purified ( Additional file 3: Figure S2) and then, exposed to maleimidylpropionyl-biocytin, which can be detected with streptavidin (see Experimental section). The Cys^12-^ protein was not modified by MPB indicating the cysteine-requirement of this alkylating reagent (last lane, Figure 7A). However, both LPCAT1 and the C^211^T protein were labeled by MBP (Figure 7A). These results confirmed the sensitivity of more than one -SH group to NEM and thus, that several cysteine residues, not only Cys-211, are the target of these alkylating agents.

To define the role of the 12 cysteines in the alkylation reaction, mutant forms embedded in the membrane, and presumably correctly folded, were exposed to MPB. Following treatment and quenching with excess amount of DTT, membrane proteins were dissolved in CHAPS and mutant forms were purified, as described in the Experimental section. The label was revealed with streptavidin and the different forms (unlabeled and labeled) were detected with an antibody reacting against the hexa-histidine tag. Unexpectedly, all 12 cysteines were alkylated and all mutant forms containing at least one cysteine residue were labeled by MBP (Figure 7B). Even the mutant enzyme C^211^T C^(216,314,443,501,514)^A, whose activity was not affected by treatment with NEM, was also labeled by MPB (Figure 7B, lane NEM^r^). This result established that alkylation of some of the 6 cysteines still present in C^211^T C^(216,314,443,501,514)^A were susceptible to alkylation and that their alteration had no effect on activity of the enzyme (Figure 5B, last bars). It cannot be ruled out that substitution of one, or more, cysteine resulted in a structural change and the exposure of thiol groups that were otherwise protected from labeling in the native form. However, the findings obtained with the C^211^T C^(216,314,443,501,514)^A enzyme established that modification of LPCAT1 by alkylating agents is due to attack of several cysteine residues that are not essential for catalysis.

## Discussion

In the calmodulin superfamily of Ca^2+^-binding proteins, repeats of EF-hand motifs are often present in tandem but few forms, such as the members of the S100 family, contain a single pair of sites [39]. Member 1 and 2 of the LPCAT family have two EF-hand motifs and thus, belong to the S100 Ca^2+^-binding protein family [16,18,28]. In the helix-loop-helix structure of such calcium-binding sites, the loop coordinates the divalent cation to seven oxygen atoms of semi-conserved residues [30-32,38]. There is a great variability of sequence but the first and last residues of the loop are almost always an aspartate and glutamate, respectively. The EFh-2 motif of LPCAT1 is lacking these two important residues [16]. It was suggested that the EF-hand motifs of LPCAT1 could be structurally different with divergent functions as compared to those present in LPCAT2 [25]. Our findings establish that activity of LPCAT1 is controlled by Ca^2+^ and that the EFh-hand motifs of LPCAT1 represent active Ca^2+^-binding sites, as in LPCAT2.

The residue found at the +1 position of motif III of LPLAT was thought to confer preference to different acceptor species (lysoPA, lysoPC or lysoCardiolipin) for acylation with acyl-CoAs. With the identification of many more members of the large LPLAT family, it now appears that presence of a cysteine, arginine or aspartate residue at that position does not define specificity to the substrate (Figure 1B). Nevertheless, these residues must play some role in catalysis since their substitution often rendered inactive forms. As shown previously by Shimizu, T and colleagues [34], we confirmed that Cys-211 of LPCAT1 could not be replaced by an arginine residue. An arginine is present at that position in the three characterized LPAAT enzymes (AGPAT1, AGPAT2 and AGPAT3) (Figure 1B). Substitution of Cys-211 to many other residues rendered inactive forms. Substitution of Arg-181 of AGPAT1 to an alanine or a lysine also rendered inactive enzymes [36]. Thus, these residues are important for activity of LPLAT enzymes but activity of the C^211^T mutant of LPCAT1 established that contrary to previous report [34], Cys-211 is not essential for catalysis. In addition, removal of Cys-211 did reduce, but did not eliminate sensitivity to NEM. It also did not prevent labeling of the protein by MPB. These results demonstrate that Cys-211 is also not crucial for the sensitivity of LPCAT1 activity to alkylating agents. The C^211^T C^(216,314,443,501,514)^A enzyme was unaffected by treatment with diamide and NEM suggesting that a combination of several cysteines among Cys-211, Cys-216, Cys-314, Cys-443, Cys-501 and Cys-514 confer sensitivity of the acyltransferase reaction to these reagent but that none of these residues is essential for catalysis.

## Conclusions

The RBC is exposed to high oxidant stress from the cytosolic side due to the hemoglobin-mediated transport of oxygen and is exposed to high concentrations of Ca^2+^ in plasma [33,46,47]. Both an increase in cytosolic calcium and oxidant stress lead to a loss of the membrane phospholipid asymmetry with the exposure of phosphatidylserine and removal of the damaged cells. The sensitivity of the de-acylation/re-acylation repair cycle suggests that the integrity of their membrane will also be compromised. In hemoglobinopathies, such as sickle cell disease and thalassemia, the presence of damaged RBCs in the circulation plays a significant aggravating role in the vasculopathy that characterizes these disorders. Thus, regulation of LPCAT1 activity might contribute to the removal of damaged-RBCs from the circulation and could represent a mechanism for the dismissal of stressed cells in other tissues.

## Methods

### Materials

[1-^14^C]C18:1-CoA (55.0 mCi/mmole) was from from Amersham Corp., (Arlington Heights, IL, U.S.A) and 1 acyl-lysoPC from Avanti Polar Lipids, Inc. (Alabaster, AL, U.S.A). TLC silica plates were obtained from Analtech. Inc. (Newark, DE, U.S.A). N-Ethylmaleimide (NEM) and diamide were from Sigma-Aldrich, Inc. (St. Louis, MO, USA). Na-(3-maleimidylpropionyl)biocytin (MPB) was from Invitrogen. All other compounds used were reagent grade.

### DNA manipulations

Cloning of mouse LPCAT1 was previously reported [16]. Full-length cDNA was cloned in pET28a vector (Novagen), with a unique in-frame hexahistidine tag at the N-terminus, to yield plasmid pFK192. Site-directed mutagenesis experiments were performed with the QuikChange Multi Site-directed Mutagenesis kit (Stratagene) according to the manufacturer instruction. Primers were designed with the QuikChange® Primer Design Program (Stratagene) and are listed in Table 1. The presence of the intended nucleotide change(s) and the absence of unwarranted mutations were verified by full-length sequencing of the constructs. Position of the amino acid residues is given relative to the full-length mouse LPCAT1 protein NP_663351 (534 amino acids).

### Protein expression, membrane preparation and detection

Expression of (His)_6_-LPCAT1 was previously reported [16]. Production of (His)_6_-LPCAT1 was obtained by growing *E. coli* strain, Rosetta™ 2(DE3) transformed with the different recombinant constructs in presence of 500 μM of IPTG for 3 hours. Cells were collected, disrupted and membrane fractions were obtained by centrifugation as previously described [16]. Samples were stored at −80°C in Tris–HCl 0.2 M pH 7.4 with 10% glycerol. For analysis, protein samples were denatured for 20 min at 37°C in SDS-PAGE loading buffer and separated on SDS-PAGE 12% gel. Proteins were visualized by staining (GelCode Blue stain reagent, Pierce) and recombinant (His)_6_-LPCAT1 proteins were detected with a commercial HRP-conjugated anti-histidine antibody (INDIA-HisProbe-HRP antibody, Pierce), diluted a thousand fold. HRP detection was performed with SuperSignal West Pico Chemiluminescent kit (Thermo Fisher Scientific Inc., Rockford, IL).

### Measurement of LPCAT activity in isolated E. coli membranes

Incorporation of ^14^C]acyl-CoA into egg lysoPC by recombinant LPCAT1 protein in *E. coli* membranes was determined as previously described [16]. Reactions were performed in glass tubes at 37°C in a shaking water bath, in 200 μl of (Tris–HCl 20 mM, pH 7.4; Tween-20 0.8 mg/ml) containing 20 μM lysoPC and 5 μM ^14^C]acyl-CoA. Reactions were initiated by addition of 1 to 15 μg of membrane protein fractions and incubated from 0 to 8 min. Three to four time points, in triplicate, were used to determine the PC formation rate by LPCAT1 enyzmes. Preliminary experiments were performed to determine the correct amount of microsomes necessary to obtain a linear dependency of PC formation by the different mutant forms ( Additional file 2: Table S1). For forms with very low activity up to 15 μg of microsomes were used in each reaction. Control experiments were performed with membrane fractions obtained from *E. coli* strains transformed with the empty pET28a vector. Under our growth condition, no detectable *E. coli* acyl-CoA: 1-acyl-lysoPC acyltransferase activity was detected (first lane of Figure 4A and Figure 6A). Reactions were stopped by the addition of 200 μl of CHCl_3_:MeOH:12 N HCl (40:40:0.26, v/v) and vigorous vortexing. Phases were separated by centrifugation at 1,000 g for 5 min and the lipid-containing chloroform phase was dried down under N_2_, and dissolved by vortexing in 20 μl of CHCl_3_:MeOH (2:1, v/v). Samples were applied to TLC silica plates and developed with chloroform/methanol/acetic acid/0.9% NaCl (100:50:16:5, v/v). TLC plates were air-dried for 20 min and exposed to a PhosphoImager screen (Storm 840, Molecular Dynamics). Quantification of PC formation was performed with ImageQuant software subtracting the plate background.

To determine the effect of divalent cations on LPCAT1 activity the rate of [^14^C]PC formation was measured in absence or presence of 10 mM calcium chloride in the incubation mixture. Assays were performed in triplicate. The samples to be compared were applied and developed on the same plate. The relative activity rate is expressed as the ratio of the rates in the presence compared to the rate in the absence of the divalent cation. To determine the effect of N-ethylmaleimide and of diamide on LPCAT1 activity membrane fractions were incubated with the chemical for 30 minutes on ice in Tris–HCl 20 mM pH 7.4. The incubation in 10 μl, was followed by a 20 times dilution into the reaction mixture (200 μl). In some assays, membrane samples were first treated with 0.5 mM NEM or diamide and one half of the mixture was then incubated with 20 mM dithiothreitol (DTT) for 20 min at room temperature. Samples were then assayed for LPCAT activity as described above.

### Metal-affinity purification of (His)_6_-LPCAT1, (His)_6_-C^211^T and (His)_6_-Cys^12-^proteins

Cells were grown and proteins were induced as described above. Proteins were purified using His GraviTrap and His buffers kit of Amersham (GE Healthcare) in presence of 1% CHAPS. Frozen cell pellets obtained from 1 liter of culture were thawed on ice in 10 ml of breakage buffer (20 mM Sodium Phosphate pH 7.4, 0.5 M NaCl, 20 mM Imidazole, 1 mM PMSF and 10% glycerol). Cells were disrupted with a French-press cell at 12,000 psi. The resulting lysate was cleared by centrifugation at 16,000 g for 20 min at 4°C. ß-Mercaptoethanol was added to a final concentration of 5 mM. Membranes were collected by centrifugation at 100,000 g for 1 hr at 10°C. The pellet was slowly suspended in 1 ml of breakage buffer containing 2% CHAPS with a stir bar on a magnetic plate for about 30 min. One ml of breakage buffer was added, and 5 ml of breakage buffer containing 1% CHAPS was then mixed to obtain 7 ml of sample in BB with 1% CHAPS. It was loading on pre-washed and equilibrated Nickel column. The sample was passed 2 to 3 times on the column to improve binding of the protein. The column was washed with 20 volume of breakage buffer containing 1% CHAPS. Stepwise elution was performed with 3 ml BB containing 1% CHAPS and 50 mM, 200 mM and 500 mM imidazole. As shown on Additional file 3: Figure S2, LPCAT1 and mutant forms were detected in the third elution with few contaminant proteins.

### MPB labeling and detection

Na-(3-maleimidylpropionyl) biocytin and all mixtures containing it were kept protected from light. MPB was dissolved in DMSO at 2 mM and kept at −20°C. Membrane fractions (25 μg) and purified proteins (2 μg) were labeled in 25 μl of 10 mMK-Phosphate pH 7.4 with 100 μM of MBP. Reactions were performed for 15 min at room temperature. Reactions were stopped by quenching un-reacted MBP with 25 mM DTT for 5 min at room temperature. For purified proteins, samples were mixed with SDS-PAGE loading buffer, denatured and separated on 12% gel SDS-PAGE. For membrane fractions, the (His)_6_-proteins were extracted and purified before analysis on SDS-PAGE and detection of the MPB label. After quenching by DTT, membranes in 25 μl were solubilized in 200 μl of buffer B (urea 7 M, NaH_2_PO_4_ 100 mM and Tris–HCl 20 mM pH8.0) for 20 min at room temperature. Samples were cleared by centrifugation at 14,000 g for 5 min. The supernatant was mixed with 50 μl of washed Ni-NTA slurry 50% and incubated for 10 min. The resin was then washed twice with buffer C (urea 8 M, NaH_2_PO_4_ 100 mM and Tris–HCl 20 mM pH6.3). The (His)_6_-protein bound to the resin was then eluted in 50 μl buffer E (urea 8 M, NaH_2_PO_4_ 100 mM and Tris–HCl 20 mM pH4.5). This extraction procedure was efficient and very little (His)_6_-proteins were lost in the insoluble pellet or in the washes. Eluted samples were then mixed with SDS-PAGE loading buffer and separated on 12% gel SDS-PAGE. When necessary, Tris–HCl 2 M pH 8.0 was used to increase the pH of the eluted sample before loading. After electrophoresis, proteins were transferred onto a PVDF membrane. The blot was blocked with 1% BSA (fraction V) in TBS with Tween-20 0.1% for 60 min at room temperature. Hexahistidine tag of the recombinants proteins was detected with an HRP-conjugated anti-histidine antibody at a 1/3,000 dilution or the MPB-label was revealed by incubation with an HRP-conjugated streptavidin ligand at a 1/6,000 dilution in TBS with Tween-20 0.1% for 2 to 4 hr at room temperature. HRP detection was performed with SuperSignal West Pico Chemiluminescent kit (Thermo Fisher Scientific Inc., Rockford, IL).

## Abbreviations

ACSL, Long-chain acyl-CoA synthetase; GPAT, Glycerol-3-phosphate acyltransferase; LPCAT, Acyl-CoA:lysophosphatidylcholine acyltransferase; LPLAT, Acyl-CoA:lysophospholipid acyltransferase; PC, Phosphatidylcholine; lysoPL, Lysophospholipid; lysoPC, Lysophosphatidylcholine.

## Competing interests

Both authors declare that they have no competing interests.

## Authors’ contributions

ES conceived the study, its design, and carried out the experiments. FK participated in the conception of the study and interpretation of data. ES and FK wrote the manuscript. Both authors read and approved the final manuscript.

## Supplementary Material

Additional file 1 **Figure S1.Acyl-transferase activity of Cysteine substitution mutant enzymes.** Mutant forms listed in Additional file 2: Table S1 and that are not shown in Figure 6 are presented. The activity rate of clone FK480 (Additional file 2: Table S1, row 5) and FK481 (Additional file 2: Table S1, row 4) and the effect of treatment by 1 mM NEM and 1 mM diamide on their activity are shown in panel A. Production of C211T C501A protein (clone FK621; Additional file 2: Table S1, row 3) and of clone FK622 (Additional file 2: Table S1, row 16) and FK623 (Additional file 2: Table S1, row 18) is shown in panel B. The activity rate of the form C211T C501A was not determined but the enzyme was active (panel C, right). Clone FK622 and FK623 do not produced detectable amount of [14C]-PC (panel C, left). (A) Activity rate measurements of LPCAT1, of C211T and of clone FK480 and FK481 were performed with 5 μM [14C]-C18:1-CoA in presence of 20 μM LPC at 37°C with 1 μg of proteins. After separation by thin-layer chromatography (as shown on panel C), the amount of [14C]-PC formed during the reaction was quantified by phosphoimaging and the activity rate values are reported as PC formed/ μg of protein per min. Values are reported relative to the activity rate obtained with LPCAT1 enzyme. Treatment with diamide (1 mM) and N-ethylmaleimide (1 mM) were performed for 30 min on ice before assaying acyltransferase activity. For each protein, value obtained in absence of treatment (buffer) was set at 1 and values obtained after treatments were calculated relative to it. Results of a representative experiment are presented. (B) Proteins were separated on a 12% gel SDS-PAGE and stained with coomassie blue. Molecular mass standard (Precision Plus Protein Standard, Bio-Rad) is shown on the left. Position of the LPCAT1 protein and of the mutant forms is indicated with a black arrow on the left. (C) Detection of the lysoPC-acyltransferase activity was performed with 5 μM [14C]-C18:1-CoA in presence of 20 μM LPC at 37°C with 2 μg of proteins for 2, 4 and 6 min. Products were separated on silica plates and detected by phosphoimaging. Position of the un-reacted substrate [14C]-acyl-CoA and of the product [14C]-PC are indicated. Click here for file

Additional file 2 **Table S1.**Cysteines substitution mutants. Click here for file

Additional file 3 **Figure S2.**Purification of LPCAT1, C^211^T and Cys^12-^ protein. Proteins were produced in *E. coli* and extracted from the membrane in presence of 2% CHAPS. Then, CHAPS concentration was decreased to 1%, and proteins were applied to a Nickel-Sepharose column. After washing of un-bound proteins, hexa-Histidine tagged LPCAT1 and mutant forms were eluted with imidazole concentration of 50, 200 and 500 mM, as indicated. Ten μl of samples were loaded of 12% SDS-PAGE gel and after separation by electrophoresis, proteins were stained with coomassie blue. Note that all three forms were partially pure (indicated by an asterisk) in the fraction eluted by 500 mM imidazole. Molecular mass standard is shown of the right side of the gels. Click here for file
