# Supplementary figures and images for: Phosphatidylcholine formation by LPCAT1 is regulated by Ca2+ and the redox status of the cell
Source: BMC Biochem. 2012 Jun 7;13:8. doi: 10.1186/1471-2091-13-8 (PMC3439698; doi:10.1186/1471-2091-13-8)

**A**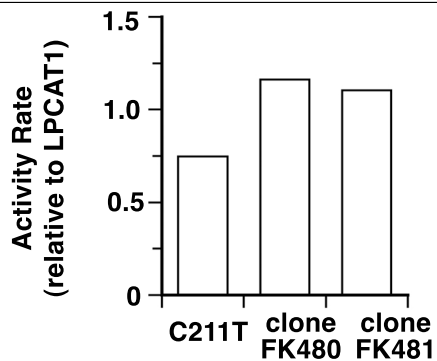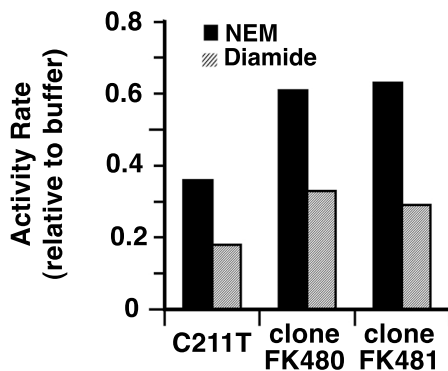**B. Protein Detection**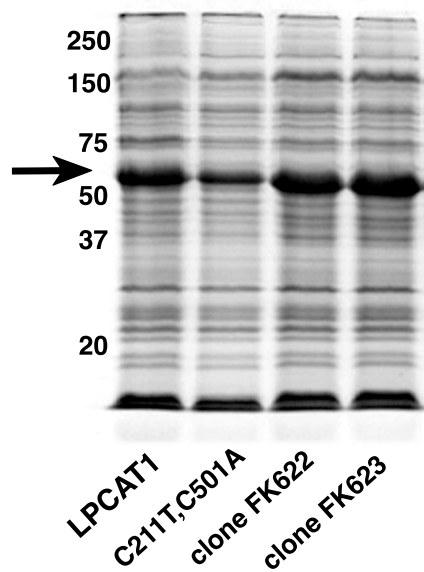**C. LPC-acylTransferase activity detection**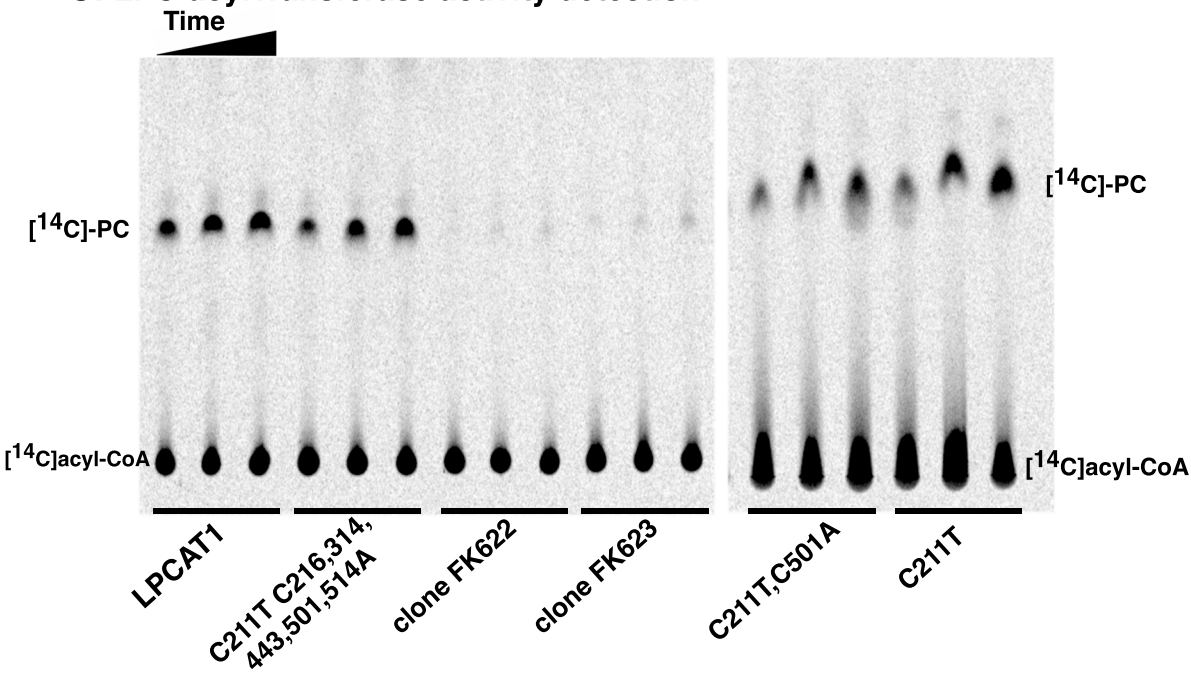

Supplement: Additional file 1 — Figure S1.Acyl-transferase activity of Cysteine substitution mutant enzymes. Mutant forms listed in Additional file 2: Table S1 and that are not shown in Figure 6 are presented. The activity rate of clone FK480 (Additional file 2: Table S1, row 5) and FK481 (Additional file 2: Table S1, row 4) and the effect of treatment by 1 mM NEM and 1 mM diamide on their activity are shown in panel A. Production of C211T C501A protein (clone FK621; Additional file 2: Table S1, row 3) and of clone FK622 (Additional file 2: Table S1, row 16) and FK623 (Additional file 2: Table S1, row 18) is shown in panel B. The activity rate of the form C211T C501A was not determined but the enzyme was active (panel C, right). Clone FK622 and FK623 do not produced detectable amount of [14C]-PC (panel C, left). (A) Activity rate measurements of LPCAT1, of C211T and of clone FK480 and FK481 were performed with 5 μM [14C]-C18:1-CoA in presence of 20 μM LPC at 37°C with 1 μg of proteins. After separation by thin-layer chromatography (as shown on panel C), the amount of [14C]-PC formed during the reaction was quantified by phosphoimaging and the activity rate values are reported as PC formed/ μg of protein per min. Values are reported relative to the activity rate obtained with LPCAT1 enzyme. Treatment with diamide (1 mM) and N-ethylmaleimide (1 mM) were performed for 30 min on ice before assaying acyltransferase activity. For each protein, value obtained in absence of treatment (buffer) was set at 1 and values obtained after treatments were calculated relative to it. Results of a representative experiment are presented. (B) Proteins were separated on a 12% gel SDS-PAGE and stained with coomassie blue. Molecular mass standard (Precision Plus Protein Standard, Bio-Rad) is shown on the left. Position of the LPCAT1 protein and of the mutant forms is indicated with a black arrow on the left. (C) Detection of the lysoPC-acyltransferase activity was performed with 5 μM [14C]-C18:1-CoA in presence of 20 μM [file 1471-2091-13-8-S1.pdf]

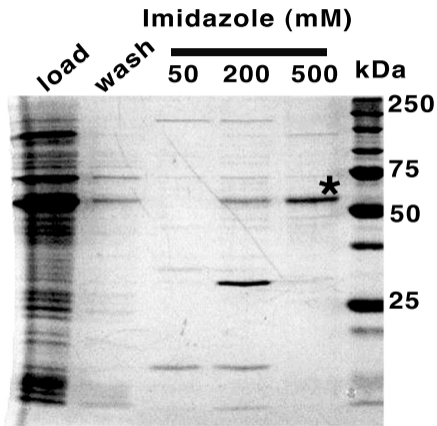

LPCAT1

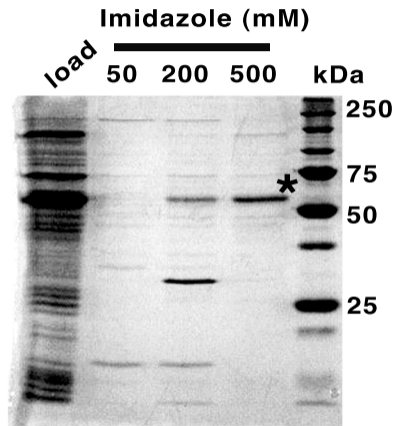

C211T

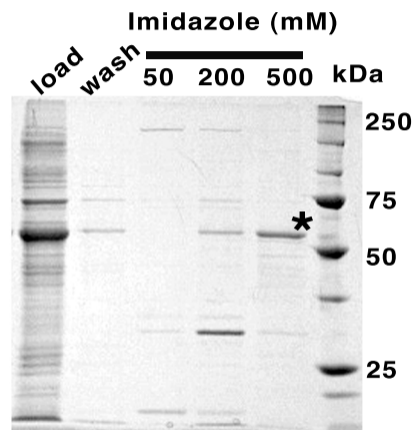

Cys12-

Supplement: Additional file 3 — Figure S2.Purification of LPCAT1, C211T and Cys12- protein. Proteins were produced in E. coli and extracted from the membrane in presence of 2% CHAPS. Then, CHAPS concentration was decreased to 1%, and proteins were applied to a Nickel-Sepharose column. After washing of un-bound proteins, hexa-Histidine tagged LPCAT1 and mutant forms were eluted with imidazole concentration of 50, 200 and 500 mM, as indicated. Ten μl of samples were loaded of 12% SDS-PAGE gel and after separation by electrophoresis, proteins were stained with coomassie blue. Note that all three forms were partially pure (indicated by an asterisk) in the fraction eluted by 500 mM imidazole. Molecular mass standard is shown of the right side of the gels. [file 1471-2091-13-8-S3.pdf]
