# Supplementary material for: Phosphatidylcholine formation by LPCAT1 is regulated by Ca2+ and the redox status of the cell
Source: BMC Biochem. 2012 Jun 7;13:8. doi: 10.1186/1471-2091-13-8 (PMC3439698; doi:10.1186/1471-2091-13-8)
Supplement: Additional file 2 — Table S1.Cysteines substitution mutants. [file 1471-2091-13-8-S2.pdf]

Supplemental Table 1. Cysteines substitution mutants

Clone	lane in Figure 6A	Protein	Activity[1]	Cysteines Position[2]											
				211	***216***	***263***	***278***	***314***	***330***	***401***	***443***	***488***	***501***	***514***	***530***
	2	**LPCAT1**	+												
FK469	3	**$C^{211}T$**	+	T											
FK621	Fig. S1	**$C^{211}T$ $C^{501}A$**	+	T									A		
FK482	4	**$C^{211}T$ $C^{443}A$**	+	T							A				
FK480	5		+	T				A			A				
FK481	6		+	T	A						A			A	
FK493	7	**$C^{211}T$ $C^{(216,314,443,501,514)}A$**	+	T	A			A			A		A	A	
FK492	8		-	T	A	A					A		A	A	
FK495	9		-	T	A	A					A			A	
FK494	10		-	T	A	A		A			A		A	A	
FK605	11		-	T	A	A		A		A	A		A	A	
FK607	12		-	T	A	A		A	A		A		A	A	
FK606	13		-	T	A	A		A	A	A	A		A	A	
FK609	14		-	T	A	A		A	A	A	A		A	A	A
FK613	15		-	T	A	A	A	A	A	A	A	A		A	A
FK622	Fig. S1		-	T	A	A	A	A	A	A	A		A	A	A
FK614	16	**Cys^{12-}**	-	T	A	A	A	A	A	A	A	A	A	A	A
FK623	Fig. S1		-		A	A	A	A	A	A	A	A	A	A	A

[1] Activity rates of the enzymes are shown in Figure 4, Figure 6 and Supp. Figure S1.

[2] Position of the 12 cysteines residues and substitution to a threonine (T) or an alanine (A) are indicated in the corresponding column.
